# The Virtual Database of the Documented Human Osteological Collection (DHOC) of the Certosa Cemetery of Bologna (Italy, 19th–20th Century)

**DOI:** 10.1002/ajpa.25065

**Published:** 2025-02-12

**Authors:** Rita Sorrentino, Annalisa Pietrobelli, Davide Mameli, Valentina Mariotti, Teresa Nicolosi, Maria Giovanna Belcastro

**Affiliations:** ^1^ Department of Biological, Geological and Environmental Sciences University of Bologna Bologna Italy; ^2^ Department of Human Origins Max Planck Institute for Evolutionary Anthropology Leipzig Germany; ^3^ Department of Cultural Heritage University of Bologna Ravenna Italy

**Keywords:** Bologna, documented collections, ethics, human skeletons, virtual anthropology

## Abstract

This article aims to introduce a new virtual database of skeletal human remains from the Documented Human Osteological Collection (DHOC) of the Certosa Cemetery of Bologna (Emilia Romagna, northern Italy) housed at the University of Bologna. The Virtual DHOC of the Certosa Cemetery of Bologna (VirtualDHOC) is stored in the publicly accessible 3D data repository MorphoSource, and consists of 3D models or micro‐Computer Tomography scans of skeletal elements of a subsample of the 425 individuals. These skeletons, mostly complete and well‐preserved, pertain to individuals of different ages, ranging from fetuses/newborns to 91 years old, for which the sex is known, who died in the city of Bologna between 1898 and 1944. This collection represents an important scientific resource for the study of human skeletal remains and to test methods and techniques in the field of bioarchaeology, forensics, and paleoanthropology. The digitization of a comprehensive database of 3D scans and 3D bones is an ongoing project that will implement the Virtual DHOC of the Certosa Cemetery of Bologna over the next few years. The Virtual DHOC of the Certosa Cemetery of Bologna aims to share the data contained therein with other researchers, contributing to the dissemination of knowledge and the promotion of scientific research in anthropology, while also ensuring the virtual preservation and accessibility of this collection for future generations. This simultaneously responds to various ethical concerns and best practices about the treatment and management of human skeletal remains.

## Graphical abstract

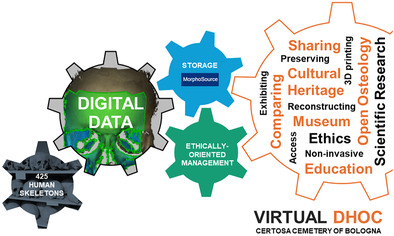

## 1 The Virtual Database of the DHOC of the Certosa Cemetery of Bologna

Human skeletal remains constitute most of the anatomical and anthropological collections stored over time and all over the world in academic and museum institutions. It is well‐known that human skeletal remains have a prominent role as evidence of human evolution, and as a comparative source for reconstructing variability, demography, and habits of past populations, as well as the life‐history of the individuals, both in archeological and forensic contexts (Belcastro, Manzi, and Moggi Cecchi 2022).

Among osteological series, compared with archaeological collections, the Documented Human Osteological Collections (DHOCs) represent unique and valuable sources because of the broad range of information they encompass, typically including data about age‐at‐death, sex, and, in some cases, occupation and cause of death of the individuals.

**FIGURE 1:**
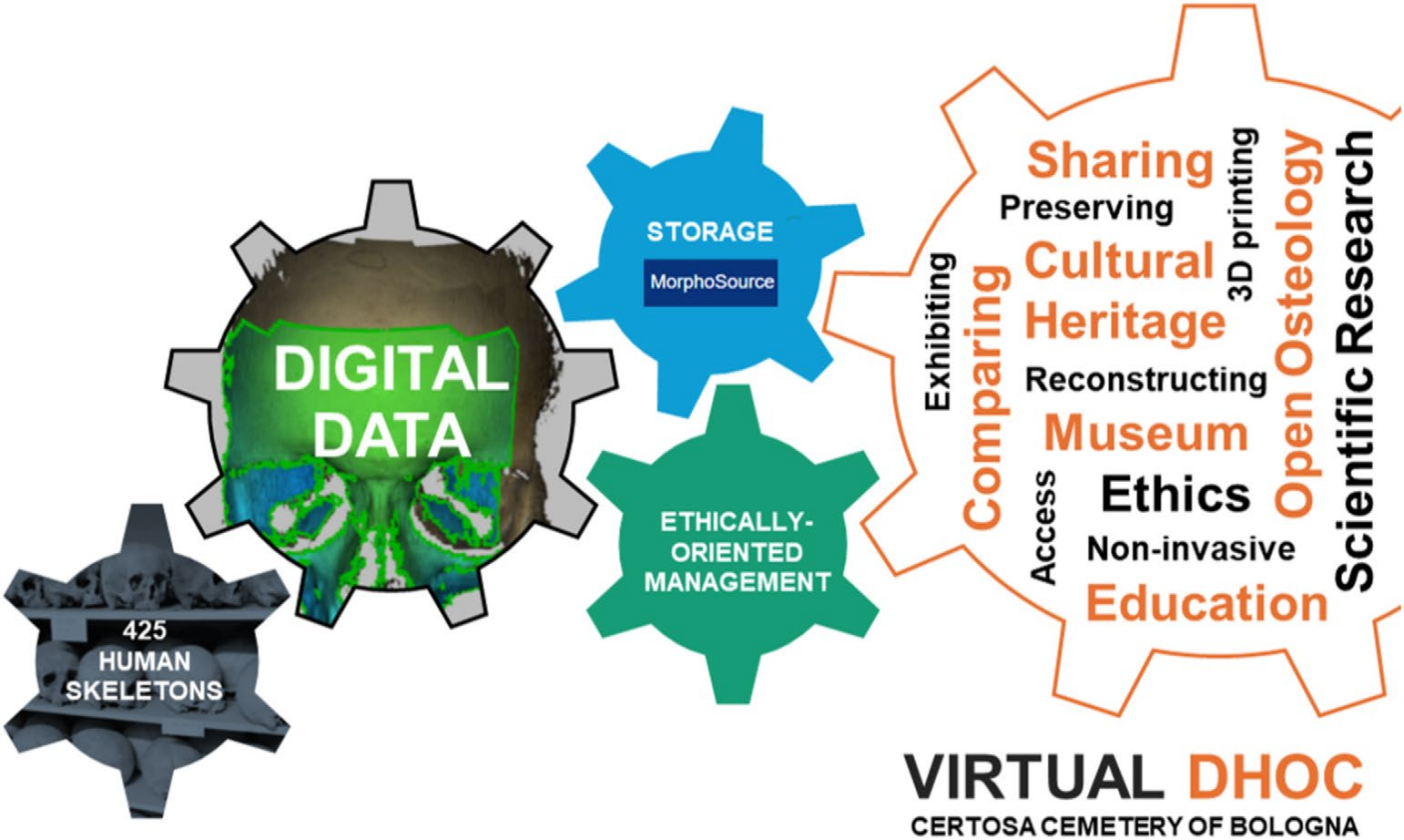
The virtual DHOC of the certosa cemetery of Bologna.

This project introduces the digitization initiative of the DHOC of the Certosa Cemetery of Bologna (Figure 1) with the broader goal of the creation of an extensive collection of 3D scans and 3D bone models available on MorphoSource (VirtualDHOC). This collection was assembled in the first half of the 20th century by Fabio Frassetto (1876–1953) and Elsa Graffi Benassi (1901–2000), at the onset of anthropology and with the aim to study the human biological variability in a hierarchical, static and racial view. With the definitive paradigmatic shift of the discipline that occurred in the last decades, these collections offer a great resource to understand the human variability in a dynamic and evolutive perspective. Today they are housed at the Department of Biological, Geological and Environmental Sciences (BiGeA) of Alma Mater Studiorum—University of Bologna and contribute to the understanding of the human evolution and variability. The DHOC of the Certosa Cemetery of Bologna consists of 425 human skeletons (mostly complete and well‐preserved) belonging to individuals deceased between 1898 and 1944 in Bologna (Belcastro et al. 2017). In compliance with the regulations for ordinary burials at the Certosa Cemetery of Bologna, the remains of ordinary burials were to be kept for a decade, after which, based on available space, they would be exhumed and transferred to common ossuaries unless private tombs were purchased (e.g., relatives had to pay more to keep them longer in the same burial location or pay to have them reburied elsewhere). Documents examined by Vidor (Vidor 2012) on the Certosa Cemetery confirmed that the enclosures from which the skeletons originate were reserved for individuals from less affluent social classes, mostly Bologna citizens, whose remains were not claimed by relatives (Belcastro et al. 2017). Currently, the human skeletal remains are preserved in wooden boxes identified with a serial number, symbols indicating the sex (i.e., ♂, ♀), and the age at death of the individual. In addition to the human remains, the collection includes the name, sex, age‐at‐death (ranging from newborns to 91 years), date and place of birth and death, occupation, cause of death, names of relatives, and, if present, spouses.

The DHOC of the Certosa Cemetery of Bologna represents a scientific standpoint for the study of human skeletal remains and to test methods and techniques in the field of archaeology, forensics, anthropology, and paleoanthropology. Furthermore, it has a cultural value as it documents the biological and demographic aspects of northern Italian populations from the late 19th to the early 20th century. Indeed, the DHOC of the Certosa Cemetery of Bologna has been studied to develop or to test traditional anthropological and virtual anthropological methods (Nelson et al. 2021; Pietrobelli et al. 2022b), for reconstructing the biological profile (Belcastro et al. 2021; Hens, Rastelli, and Belcastro 2008; Marino et al. 2021; Milella et al. 2020; Pietrobelli et al. 2022a; Sorrentino et al. 2020a; Viciano et al. 2021), paleopathological conditions (Sorrentino et al. 2022; Mariotti et al. 2015; Zampetti et al. 2016), ontogenetic studies (Belcastro et al. 2019; Colombo et al. 2019; Figus et al. 2022, 2023b; Mameli et al. 2024; Oxilia et al. 2021; Pietrobelli, Marchi, and Belcastro 2022), and as a reference for the contemporary human variation in several anthropological and paleoanthropological researches (Belcastro et al. 2020; Belcastro and Mariotti 2017; Pietrobelli et al. 2023; Sorrentino et al. 2020c, 2021, 2023). Over the years, most of the above‐cited studies generated N°326 3D models and N°126 micro‐Computed Tomography (CT) scans of skeletal elements that have not yet been made publicly available.

The Virtual database of the DHOC of the Certosa Cemetery of Bologna (VirtualDHOC) aims at making the digital visual database of DHOC skeletons easily accessible, allowing researchers, students, and museum staff to access a digital resource that extensively documents the cranial and post‐cranial morphology of individuals who lived from the late 19th to the early 20th century in Bologna. Thanks to the CHANGES project (Cultural Heritage Active Innovation for Sustainable Society, Spoke n. 6 “History, Conservation and Restoration of Cultural Heritage” of the Piano Nazionale di Ripresa e Resilienza—PNRR) financed by the European Union—Next Generation EU (CHANGES) all these media will be now accessible on MorphoSource (VirtualDHOC). Efforts will be made to secure additional funding from the same sources, as well as exploring other potential funding opportunities, in order to continue the project and ensure its long‐term maintenance. The University of Bologna will remain responsible for managing the database, including the integration of new models and responding to research enquiries. MorphoSource has been chosen as it represents a stable platform in which databases of 3D models can be uploaded, stored, and updated under the supervision of the institutional and scientific referent of the collection. It was selected due to its widespread use in the research community, offering reliable data storage, long‐term accessibility, and regular updates to ensure data integrity. Importantly, there are no costs associated with storing and accessing data. The work carried out represents an initial phase of a broader digitization project aimed at digitizing various skeletal areas of the individuals belonging to the collection, which consists of more than 425 digital media that come in part from edited research studies listed below. Currently, the virtual database contains (Table 1):

**TABLE 1:** Summary content of DHOC by number, file type and skeletal element.

Number of scans	Type	Description	References
49	3D model	Crania of adult individuals	
53	3D model	Mandibles of adult individuals	
44	3D model	Fibulae of adult individuals	Pietrobelli et al. 2022a, 2022b; Pietrobelli et al. 2023
50	3D model	Tali of adult individuals	Sorrentino et al. 2020a, 2020b, 2020c; Sorrentino et al. 2021, 2022
8	Micro‐CT	Tali of adult individuals	Sorrentino et al. 2020a, 2020b, 2020c; Sorrentino et al. 2021, 2022
52	3D model	Naviculars of adult individuals	Sorrentino et al. 2023
51	3D model	Calcanei of adult individuals	Serrangeli 2021
14	Micro‐CT	Tali of non‐adult individuals	Figus et al. 2022; Figus et al. 2023a, 2023b
11	Micro‐CT	Calcanei of non‐adult individuals	
27	3D model	Ilium of non‐adult individuals	
50	Micro‐CT	Femur distal metaphysis of non‐adult individuals	Colombo et al. 2022a, 2022b
43	Micro‐CT	Radii distal metaphysis of non‐adult individuals	Colombo et al. 2019; Colombo et al. 2022a, 2022b, 2022c

Table S1 lists the already available digital data, together with relevant biological information of the related individuals. Scan parameters for each digital data are available on the online resource MorphoSource (VirtualDHOC). Data stored are raw data stack and/or derived 3D models. Data size ranges from a few dozen megabytes for scanner‐generated 3D models to more than 30 gigabytes for micro‐CT generated stacks. Additional digital data will be made available in subsequent years to compile a comprehensive database of all human skeletal elements. This database serves as a crucial modern comparative sample and may also represent a focal point for future archaeological, forensic, anthropological, and paleoanthropological research in Virtual Anthropology.

## 2 Virtual Anthropology as Research Resource and Bio‐Cultural Archive

Over the last two decades, anthropologists rely more and more on advances in 3D imaging technologies with research applications of 3D digitization in human remains, such as documenting crime scenes and archaeological sites (Fernández‐Lozano et al. 2017; Soler, Melero, and Luzón 2017), conducting virtual autopsies (Bolliger et al. 2008), and performing morpho‐functional analyses to explore evolutionary and adaptive variations in extinct and extant hominins (Benazzi et al. 2011; Cook et al. 2021; DeSilva et al. 2013; Fernández et al. 2018; Kivell 2016; Shaw and Ryan 2012; Sorrentino et al. 2020c, 2023; Su and Carlson 2017; Tsegai et al. 2018; Zeininger et al. 2016).

The application of 3D technologies in the anthropological field is known as Virtual Anthropology (hereinafter “VA”), a term coined in the late 1990s. The emergence of this discipline has brought significant advantages over traditional study approaches, which adopt a direct approach to the study material (Weber 2015). VA allows the study of not only the external surface of a bone using surface virtual acquisition techniques (i.e., photogrammetry, structured light scanning, or laser scanning), but also internal structures through tomographic techniques (i.e., CT, micro‐CT, and radiography scans) that enable non‐destructive dissection (Blackburn et al. 2024; Weber 2015; Profico et al. 2016, 2019; Riga et al. 2022). These methods not only enable the visualization of internal structures, but also allow the estimation of no longer present structures, such as in the case of the brain (Carlson et al. 2011; Gunz et al. 2019). VA has facilitated the advance of geometric morphometric methods used to quantify the differences among the shapes of organisms through the statistical analysis, shedding light on the processes involved in their evolution, ontogeny, phylogeny, and adaptation (Gunz and Mitteroecker 2013; Mitteroecker et al. 2013; Mitteroecker and Gunz 2009; Slice 2005).

In addition, VA has brought tangible advantages not only in terms of research applications (e.g., morphometric analysis applied to taxonomic identification, advanced bioengineering modeling for the reconstruction of bone structural properties), but also in the broader context of conservation, data sharing, and the valorization of human remains (Weber 2015).

Within the context of conservation, several museums have promoted the creation of digital archives stored within their institutional repository (e.g., Smithsonian 3D collections, Musée de l'Homme ‐ MNHN) or by using publicly accessible 3D data repository (e.g., MorphoSource, The Human Fossil Record). Digital conservation of human remains is paramount when dealing with frail or damaged bones. Indeed, VA mitigates the risk of damage and deterioration of bones as it reduces their handling during study. Virtual reconstruction is also necessary when the shape and structure of fragile elements need to be analyzed, but the current preservation is altered due to taphonomic processes. Methods for virtual reconstruction of incomplete and/or damaged human remains involve estimating missing parts using geometric morphometric methods or software‐guided alignment and symmetrization of isolated fragmented parts (Benazzi et al. 2011; Ni, Flynn, and Wyss 2012; Schlager et al. 2018; Sorrentino, Belcastro et al. 2020; Sorrentino et al. 2020b; Veneziano, Landi, and Profico 2018; Weber 2001). Storing virtual copies of human remains can also allow access to temporarily exhumed human remains that have to be reburied, as in the case of the digitization initiative of the famous 18th century singer Farinelli, undertaken by research institutions for analytical purposes, and abiding by the national regulations (Belcastro et al. 2014). VA also provides the possibility to digitally acquire and analyze skeletal elements that are inaccessible due to the peculiar context of their retrieval, as in the case of the Neanderthal cranium from Altamura in Italy (Profico et al. 2023).

Regarding the context of data sharing, VA has allowed researchers to expand their professional network worldwide, facilitating the collaboration of international research communities on large projects, even in extremely challenging situations, such as that experienced during the Covid‐19 pandemic. These practices were indeed encouraged by a broader paradigmatic shift toward open science, which calls researchers to share openly published digital datasets and data in anthropology, with unique benefits and a range of challenges (Mulligan et al. 2022; Turner and Mulligan 2019). Storing and sharing virtual copies requires considering the platform, the size of the generated, who would have access permissions (researchers, educators, and artists), and how those permissions would be granted (agreements, restricted permissions, free availability). In most circumstances, data accessibility is governed by the museum or university where the physical data are stored.

Regarding the context of museum display, digitization has the advantage of allowing the creation of online virtual collections accessible to visitors. Furthermore, it offers the possibility of developing interactive paths through the creation of physical casts with 3D printers, offering visual contents in the physical space of the display and tactile itineraries even for visually impaired people (Riga et al. 2022). Additionally, it enables the creation of digital databases that ensure accessible information to a wider audience, promoting participative culture. While 3D web viewer skills are common in research, they are also becoming widespread among diverse user communities. VA also addresses issues related to the exhibition of human remains, considered culturally sensitive materials, overcoming the challenges related to the physical exhibition while allowing demonstration of the digital copy (Belcastro, Manzi, and Moggi Cecchi 2022; Belcastro et al. 2022; Riga et al. 2022). A recent on‐line survey involving Portuguese residents highlighted a general agreement on the creation, access, use, and dissemination of 3D digital models of human bones (Alves‐Cardoso and Campanacho 2022).

Still, as far as we know, internationally standardized guidelines for the digitization of bio‐cultural anthropological heritage are lacking. As regards Europe, 27 countries signed the Declaration of Cooperation on Advancing the Digitization of Cultural Heritage in 2019, leading to the 10 fundamental principles for the 3D digitization of cultural heritage (European Commission 2019). These 10 points cover assessing the value of 3D digitization, selecting items to digitize, deciding between in‐house or outsourced digitization, addressing copyright and access, ensuring quality standards, identifying necessary formats, planning for long‐term preservation, using appropriate equipment and methods, protecting assets, and investing in 3D technology knowledge. This declaration is a pan‐European initiative to 3D digitize monuments, sites, and cultural assets, and to invest in knowledge of 3D technologies, processes, and contents. Furthermore, the European Union supports research and innovation through the framework of the Horizon Europe program, an initiative aimed at creating a European collaborative network dedicated to the digitization of cultural heritage through grants. This program supports digitization initiatives by consolidating them, thus contributing to the preservation of European heritage (HorizoneEurope2023). In this context, the Central Institute for the Digitalization of Cultural Heritage of the Italian Ministry of Culture has drawn up a National Plan for the Digitalization of Cultural Heritage, mainly aimed at museums, archives, and libraries, building from the European initiatives. This plan defines guidelines for the digitization of cultural heritage, establishing procedures for creating virtual assets, data management, acquisition, circulation, and reuse (PND).

Building upon research advances facilitated by VA and the growing open science practices in anthropology, we aim to address the European and Italian call to 3D digitization of cultural heritage. This includes the project of digitization of the DHOC of the Certosa Cemetery of Bologna to ensure the preservation, valorization, dissemination, and study of this unique cultural asset. Additionally, we promote an ethically‐conscious approach to this digitization project, considering the sensitive aspects and the still unclear place of human remains of scientific interests in the frame of Cultural Heritage law (Legislative Decree of January 22, 2004, No. 42; Ministero della Cultura 2004), mainly due to their symbolic value from a social, cultural, and spiritual perspective (Belcastro and Mariotti 2021).

## 3 The Ethical Dimensions of Human Skeletal Remains Within the Legal Framework in Italy

Skeletal remains included in osteological collections provide a vital resource for researchers and are well recognized as invaluable bio‐cultural archives by the academic community. In Italy, even though the human remains may be included in the legal frame of cultural heritage, as they bear historical testimony of past populations, they are not specifically mentioned in the most important Cultural Heritage law (the Italian Code of Cultural and Landscape Heritage of 2004; Ministero della Cultura 2004) that defines cultural heritage as material and immaterial goods reflecting history, culture, art, and traditions of a community. Actually, an explicit set of guidelines about the management of human remains come from the Italian National Mortuary Police Regulation (D.P.R.n.285/90), also in reference to collection and use of human skeletal remains for scientific purposes. Furthermore, local authorities (e.g., municipalities) may dispose of specific regulations, particularly for the exhumation concerning available space and needs of local communities. In spite of this, the legal status of human remains in Italy is often relegated to a gray‐area likely because of their sensitive nature that eludes a clear property assignment (Belcastro, Manzi, and Moggi Cecchi 2022; Belcastro and Mariotti 2021; MiC 2022).

While only recently this topic has been approached in Italy at an academic level, guidelines and recommendations have already been proposed in USA (Cardoso 2021; Lee 2021; Lippert and Sholts 2021; Roseman 2021; Smocovitis 2021; Squires, Roberts, and Márquez‐Grant 2022; Valeggia and Fernández‐Duque 2022) and in Northern Europe as well, since the early 2000s. The American Association of Physical Anthropology (AAPA) in 2003 (AAPA, 2003), the Human Tissue Act in 2004 (Human Tissue Act 2004), and the British Association for Biological Anthropology and Osteoarchaeology (BABAO) in 2010, updated in 2019 (BABAO Guidelines, 2019), proposed detailed recommendations for best practices to adequately operate in this field. Also for the England, Wales and Northern Ireland there is the Guidance for the Care of Human Remains in Museums and other institutions that hold human remains in permanent collections (Department for Culture, Media and Sport, 2005).

In this frame, a recent handbook has been implemented by the archaeologists and anthropologists of the Italian Ministry of Culture and some academic anthropologists (MiC 2022). In addition, on the occasion of the 25th Congress (Turin, 6–8 September 2023), the Italian Anthropological Association (AAI) also promoted the creation of an interdisciplinary working group (academic biological and forensic anthropologists, anthropologists and archaeologists working at the Ministry of Culture, and the head of the Egyptian Museum in Turin) that are working for proposing guidelines for the Italian State for the management of the human remains in the scientific, educational, and museum display context.

In 2020, the Italian Government ratified the Faro Convention (FaroConvention), enacted by the European Union in 2005, allowing communities to attribute value to specific aspects of cultural heritage that they wish to support and passing on to future generations (Gualdani 2020). In this context, museums and academic institutions play a fundamental role in highlighting the cultural and scientific value of the anthropological collections, as well as in promoting them as cultural and natural heritage in research projects, education paths, and public exhibitions.

In 2009, the Code of Ethics for Museums (ICOM, 2009), to which Italian museums adhere, stated that the exhibition of sensitive assets, such as human remains and sacred materials, requires compliance with professional standards and respect of the interests and beliefs of the relevant ethnic or religious stake‐holding communities of origin. Thus, human remains management and display may raise ethical issues much more than other cultural assets. However, the unclear legal status and the lack of clear shared guidelines for the human remains, particularly for the human osteological collections stored in museums and anthropological laboratories, significantly contribute to the uncertainty in their management when ethical issues emerge.

In Italy, the archaeologically retrieved human remains of the medieval Jewish cemetery of Via Orfeo (Bologna) represented the only successful restitution in the country in 2017, when most of the over 300 skeletons were reburied (Belcastro and Mariotti 2021). Instead, requests of repatriation of human remains remained unsuccessful, such as the one moved from the Australian Government to the Anthropology and Ethnology Museum of the University of Florence, asking for the repatriation of Aboriginal remains. In response to this request, the National Association of the Scientific Museums published in 2011 a monographic volume, stressing the scientific value of human remains, but also the importance of a close collaboration with those native communities (Associazione Nazionale Musei Scientifici 2011).

In the last few years, growing worldwide efforts have been made to digitize human remains in museum displays, as a strategy to mitigate the potential risks inherent to curating and showcasing such sensitive heritage, altogether ensuring its dissemination for research purposes (Biers 2019). An example is the display of the CT scanned mummified remains of the ‘Gebelein Man’ at the British Museum (Antoine and Ambers 2014). In Italy, similar efforts were undertaken by the Museo Egizio in Turin, which vastly relies on 3D technologies for the display of human remains, such as in the permanent exhibition room ‘In search of life’ (MuseoEgizio,‘InSearchOfLife’).

Several 3D digitization initiatives involved several osteological collections and databases, with the twofold aim of accounting for creating more robust datasets for biological studies, and simultaneously protecting the sensitive nature of such data through the virtual approach. Notable examples include the project of digitization of the human remains held at the Smithsonian Institutions (Smithsonian3Dcollections), the Digitized Diseases, an initiative to create a digital archive of 1600 paleopathological specimens from archaeological collections in the UK (DigitizedDiseases), and 3D data repositories that collect digital visual database coming from different institutions and universities (MorphoSource, TheHumanFossilRecord). Other valuable resources are represented by the New Mexico Decedent Image Database, which contains full body CT‐scan images paired with ante‐mortem data of known individuals (Edgar et al. 2020), and 3D and 2D digital data of contemporary skeletal remains (Algee‐Hewitt 2016; Mallett and Evison 2017; Verhoff et al. 2008). Besides digital visual databases, there are also osteological digital datasets from analysis of archaeologically derived human skeletal remains, such as the Wellcome Osteological Research Database (WORD) held at the Centre for Human Bioarchaeology—London Museum, and anthropometric datasets from living humans, such as the Denver longitudinal Growth Study consisting of measurements taken from radiographs (Ruff 2022).

However, the process of 3D digitization of human remains poses its own challenges concerning the ethical, legal, and logistical implications (Margoni 2014; Márquez‐Grant and Errickson 2017; Thompson 2017), due to a lack of proper ethical or legal guidelines (Algee‐Hewitt 2016; Alves‐Cardoso and Campanacho 2022) and also it faces with new questions for the decolonization (Grechi 2021), a recent and ongoing process that involves also the Italian museums where anthropological and ethnographic collections are curated (Nicolosi, Battilani, and Belcastro 2024).

Although there is some early research discussing the ethics and ownership of these different data forms (Carew et al. 2023; Márquez‐Grant and Errickson 2017; Thompson 2017), there is no comprehensive consensus on crucial issues such as data ownership and access regulation to the digitized remains with specific terms of use agreements (Alves‐Cardoso and Campanacho 2022).

The ethical sharing of digitized human remains starts from a clear definition of the unclarified ownership issue. “Who has permission to share these data, how these data should be shared, who may access these data sets, and once these data are shared, how may these data be utilised by other researchers?” (Smith and Hirst 2019). However, the lack of straightforward answers to these questions has been highlighted along with the variable attitudes of researchers in managing the digitized human skeletal remains (Alves‐Cardoso and Campanacho 2022; Smith and Hirst 2019). Many of these questions are only recently emerging in Italy, as said before, and the researchers often may reply to these issues case‐by‐case. Considering this complex scenario and the importance of preserving the DHOCs in the face of possible emerging issues, their digitization would be a tentative application of the best practices for managing human skeletal remains. This approach requires a respectful and considerate attitude toward human remains, considering the ethical, scientific, cultural, and social implications surrounding this unique research resource.

The management of the DHOC of the Certosa Cemetery of Bologna adheres to the principles of modern research ethics (Belcastro, Manzi, and Moggi Cecchi 2022; Belcastro et al. 2022). The authors did not communicate with living descendants of the individuals involved in the project because of the impossibility of identifying them. The individuals involved in the study died around 100 years ago, while the collection of their remains occurred only a few decades later following ordinary cemetery procedures. Because of this, the local community was not involved in their excavation and collection, which were more likely performed by the cemetery personnel. Given the impossibility of seeking proper informed consents, according to the principles of modern research ethics following the Nuremberg Code of 1947 (Caffell and Jakob 2019; Turner, Wagner, and Cabana 2018), stringent measures are in place to handle and utilize these remains with the utmost care, respect, and dignity (e.g., regulated physical access, withholding of sensitive personal information), in adherence with national and international scientific recommendations in dealing with human remains (MiC 2022; Turner, Wagner, and Cabana 2018; BABAO Guidelines, 2019; AAPA, 2003; EC 2013).

Our ethically‐oriented initiative is driven by the goal to align research advancements in the field of VA, within the wider framework of open science principles and institutional mandates advocating for the 3D digitization of cultural heritage. We firstly recognize that digitized human skeletal remains do not have an owner, and therefore curators are responsible for making them freely available (e.g., CC BY‐NC, non‐commercial licensing), while also ensuring the protection of sensitive data (e.g., ensuring anonymity). The curators will ensure that specific terms of use conditions are met upon requesting the material. These include access application forms, terms and conditions of use, reproducibility of images, allowed 3D‐printing only for didact and scientific purposes, and so forth. The authors are perfectly aware that human remains must be handled and employed in didactic and research activities ensuring full care, respect, and dignity. As such, they guarantee that the study has been conducted according to the most advanced ethical and scientific principles for the study of human remains. Such conditions include the adherence to the national and international scientific recommendations in dealing with human remains with due respect and evaluation of the proposed research project, which should comply with the relevant scientific standards (MiC 2022; Squires, Roberts, and Márquez‐Grant 2022; Turner, Wagner, and Cabana 2018; BABAO Guidelines, 2019; AAPA, 2003; EC 2013).

In conclusion, this project marks the initial phase of an ambitious digitization endeavor, supported on the long term by the University of Bologna building upon concerns regarding the vulnerability of human skeletal remains from both biological and ethical perspectives within the bio‐cultural and scientific contexts. By openly sharing the digitized data of the DHOC of the Certosa Cemetery of Bologna, our driving mission is to contribute to the dissemination of knowledge and the promotion of scientific research in anthropology, while also ensuring the virtual preservation and accessibility of this collection for future generations.

## Author Contributions

**Rita Sorrentino:** conceptualization (lead), data curation (equal), funding acquisition (equal), investigation (equal), methodology (equal), project administration (lead), resources (equal), software (equal), visualization (equal), writing – original draft (equal), writing – review and editing (equal). **Annalisa Pietrobelli:** conceptualization (equal), data curation (equal), investigation (equal), methodology (equal), software (equal), visualization (equal), writing – original draft (equal), writing – review and editing (equal). **Davide Mameli:** data curation (equal), investigation (equal), methodology (equal), software (equal), writing – review and editing (equal). **Valentina Mariotti:** investigation (equal), validation (equal), writing – review and editing (equal). **Teresa Nicolosi:** conceptualization (equal), data curation (equal), investigation (equal), methodology (equal), validation (equal), visualization (equal), writing – original draft (equal), writing – review and editing (equal). **Maria Giovanna Belcastro:** conceptualization (lead), data curation (equal), funding acquisition (equal), investigation (equal), methodology (equal), project administration (equal), resources (equal), supervision (lead), validation (equal), writing – original draft (equal), writing – review and editing (equal).

## Ethics Statement

In Italy, including the University of Bologna, ethical boards are not consulted when studying archaeologically or cemetery‐collected human skeletal remains for scientific purposes. Nevertheless, the offices of the Department of Biological, Geological and Environmental Sciences of the University of Bologna, where the collections are hosted, were consulted to determine the best strategies to achieve the aims of this study. This study aims to make the Documented Human Osteological Collections of the University of Bologna available to other researchers within the framework of Open Science. These strategies include access application forms, terms and conditions of use, reproducibility of images, citing literature, and so forth. The authors are fully aware that human remains must be handled and used in educational and research activities with full care, respect, and dignity. They guarantee that the study was conducted according to the most advanced ethical and scientific principles for the study of human remains. The study was performed in accordance with Italian law and followed national and international institutional guidelines and regulations.

## Supporting information


**TABLE S1.** Available digital data and associated biological information of DHOC (up to 2024).

## Data Availability

The data are available upon request from MorphoSource at the DHOC of the Certosa Cemetery of Bologna. This is an ongoing project, and we are still digitizing human skeletal elements from the DHOC. To assist researchers interested in the DHOC, we prioritize scans of skeletal elements that researchers intend to use for their projects. If scholars are interested in particular skeletal elements that are not yet available on MorphoSource, please email the corresponding authors to request them.
